# Antioxidant Activities and Total Phenolic Content of Malaysian Herbs as Components of Active Packaging Film in Beef Patties

**DOI:** 10.3390/antiox8070204

**Published:** 2019-07-02

**Authors:** Wan Amnin Wan Yahaya, Noraziah Abu Yazid, Nurul Aini Mohd Azman, María Pilar Almajano

**Affiliations:** 1 Faculty of Chemical and Natural Resources Engineering, University Malaysia Pahang, Lebuhraya Tun Razak, Pahang 26300, Malaysia; 2 Chemical Engineering Department, Technical University of Catalonia, Avigunda Diagonal 647, 08028 Barcelona, Spain

**Keywords:** semi-refined carrageenan, lipid oxidation, active packaging film, antioxidant activities

## Abstract

Active packaging containing natural extracts is a promising innovation to prolong the shelf life of perishable food. The objective of this work was to develop a bioactive edible film from semi-refined carrageenan (SRC) and glycerol (G) as plasticizer incorporated with natural extract. Five Malaysian herbs were evaluated to determine their total phenolic content (TPC) and antioxidant activities. The *Persicaria minor* (PM) extract demonstrated the highest TPC (1.629 mg GAE/L sample) and radical scavenging activity evaluated by the radicals 2,2’-azinobis [3-ethylbenzothiazoline-6-sulfonic acid] (27.166 mg TE/L sample), 2,2-diphenyl-1-picrylhydrazyl (719.89 mg eq. Trolox/L sample) and α,α′-Azodiisobutyramidine dihydrochloride (5.81 mg TE/L sample). Thus, PM extract was selected for active packaging film at concentrations of 0.4, 1.0 and 2.0% and compared with 0.4% Butylatedhydroxianisole in 2% SRC and 0.9% G film formulation. The meat patties were wrapped in the films and stored under refrigeration (4 ± 2 °C) for 14 days. The film with 2% PM exhibited significantly lower lipid deterioration analysed by the thiobarbituric acid reactive substance assay (*p* < 0.05) and small changes in % metmyoglobin value which indicated the minimum development of brown colour (*p* < 0.05). Hence, this film can be used as a packaging material to improve meat quality characteristics.

## 1. Introduction

High oxidation rates are observed in animal products. The deterioration in quality of these products is highly correlated with lipid oxidation rates due to their high content in unsaturated lipids. The oxidation of lipids in foodstuffs results in the development of off-flavours, rancidity and the modification of texture and colour, and leads to the growth of microorganisms and losses of vitamins [1]. According to previous literature, toxic aldehyde formation and loss of nutritional value due to the degradation of polyunsaturated fatty acids (PUFAs) is caused by lipid oxidation. To minimize lipid oxidation, many strategies have been employed [2], such as the direct incorporation of antioxidants into foodstuffs or the design of appropriate packaging technology, i.e., vacuum or modified-atmosphere packaging combined with high-barrier packaging materials [3]. Commonly, the conventional packaging system includes butylated hydroxyanisole (BHA) and butylated hydroxytoluene (BHT) as synthetic antioxidants even though it has been claimed that these compounds can pose a potential risk to human health [4,5]. There are significant concerns related to their possible toxicity and the potential health risks caused by such compounds. Therefore, to prolong food shelf life, a promising approach is to combine natural antioxidants and biodegradable films into a single food packaging formula [6].

From a safety and marketing perspective, biopolymer films incorporating natural antioxidants are more attractive to consumers. Several studies have showed that the use of natural antioxidant extracts from galangal [7], pumpkin [8], durian leaf [9] and pequi [10] with biopolymers are able to inhibit the oxidative deterioration and retain the freshness in muscle foods. Furthermore, there are several reports that natural antioxidants that contain phenolic compounds including green tea [11], kiwi fruit [12], barley husk [13], rosemary and oregano extract [14] can not only reduce lipid oxidation in meat but also extend the functional life of the film.

Malaysian herbs such as *Cosmos caudatus* (CC)*, Piper sarmentosum* (PS), *Persicaria minor* (PM), *Centella asiatica* (CA) and *Syzygium polyanthum* (SP) are local plants that contain natural antioxidants and are consumed directly or are used in various local recipes to enhance the flavour of the foods. Recent studies have shown that CC extract possesses free radical scavenging activities resulting in intense antioxidant effects [15]. *Cosmos caudatus* has various medicinal properties, including antidiabetic, antihypertensive, anti-inflammatory, bone protective and antimicrobial activity [16]. Flavonoid components in the lyophilized CC extract include quercitrin, catechin, and rutin [17]. Previous studies have reported that PS extract possesses many pharmacological benefits including antibacterial [18], anti-inflammatory [19,20,21] and antioxidant activities [22,23]. *Piper sarmentosum* leaf extracts are reported to contain phenolic acids and flavonoids including quercetin and naringin [24]. Previous literature reported that PM leaves have high concentrations of natural plant antioxidants which can reduce the oxidative harm caused by the effects of accumulated free radicals. Nanasombat and Teckchuen [25] reported that PM includes flavonoids such as rutin, catechin, quercetin, isorhamnetin and kaempferol which act as natural antioxidants in controlled release films to minimize fatty food simulant oxidation [26]. In a recent study, preliminary screening of various medicinal plants in Malaysia showed that PM had strong antioxidant properties that are more intense compared to the synthetic antioxidant at 90% of the concentration [27]. Furthermore, CA contains high concentrations of pentacyclic triterpenoids including asiaticoside, brahmoside, asiatic acid, brahmic acid (madecassic acid) and also includes centellose, centelloside and madecassoside [28]. The main application of CA has been in the treatment of wounds. *Syzygium polyanthum* is commonly known as “salam” leaves and it is used for seasoning food because of its rich aroma. *Syzygium polyanthum* has also been widely used in Indonesian traditional medicines. It is also known to provide several health benefits because it has a wide range of bioactivity including antihypertensive, antimicrobial and antidiarrheal effects [29].

However, this Malaysian herb’s antioxidant activity and ability to retard lipid deterioration has not been studied in detail. Therefore, our objectives were (1) to evaluate the antioxidant activity of Malaysian herbs, assessed by the TEAC, ORAC, DPPH and TPC assays and (2) to determine the potential of selected natural extracts that possess intense antioxidant activity to inhibit lipid deterioration in meat patties. Hence, active packaging films prepared from 2% (w/w) semi-refined carrageenan (SRC) plasticized with 0.9% (v/v) glycerol (G) and containing natural extracts were developed to improve lipid oxidative stability and storage quality of meat patties stored for 14 days at 4 °C.

## 2. Materials and Methods

### 2.1. Plant Material and Chemicals

Five types of herbs (*Cosmos caudatus* (CC), *Piper sarmentosum* (PS), *Persicaria minor* (PM), *Centella asiatica* (CA) and *Syzygium polyanthum* (SP)) were obtained from a local supplier in Gambang, Pahang. Semi-refined carrageenan was prepared from *Eucheuma Cottonii* (Malaysian seaweed). All chemicals used were purchased from Sigma-Aldrich (Gillingham, England).

### 2.2. Preparation of Leaf Extract

The leaves of five different herbs were cleaned and dried in an oven at 30 °C for 24 hours. The dried leaves were ground using a food processor (Moulinex, France). The fine ground powder (3 g) was extracted with an aqueous solvent containing 75% ethanol at a ratio 1:20 (w/v) based on the dry weight of the ground plant. All extraction was performed in an incubator shaken at a speed of 150 rpm at room temperature for 24 hours. After 24 hours, the extraction solution was centrifuged at a speed of 10,000 rpm and a temperature of 10 °C and supernatants were separated. The extract was stored at −20 °C until further use. 

### 2.3. Determination of Phytochemical Content

The total phenolic content was analysed using the Folin–Ciocalteu method. An appropriate dilution of herbal extracts with Folin–Ciocalteu reagent, 80 μL, and sodium carbonate, 2% (w/v), was homogenized. Finally, Milli-Q water was added to the mixture for dilution. The mixture was agitated in a dark place for 1 hour. The UV-visible spectrophotometer model PharmaSpec 1700 (Shimadzu, Japan) was used to measure absorbance at 765 nm with Milli-Q water as a blank. Gallic acid was used as a standard and the results were expressed as equivalents in mg GA/L sample.

### 2.4. Determination of Free Radical Scavenging

The free radical scavenging activities of natural leaf extracts were determined using the DPPH assay, ORAC assay and TEAC assay according to the methods described previously. 

#### 2.4.1. DPPH Assay

The scavenging of DPPH radicals was quantified using a method previously described with slight modifications [30]. The DPPH assay is simple and fast because a DPPH radical is a relatively stable organic nitrogen radical which is commonly used in antioxidant testing [30]. The reduction of the purple chromogenic DPPH radical to the corresponding pale-yellow hydrazine by an antioxidant or a reducing compound [30] determines the extract scavenging ability. The DPPH reagent (0.1 mM) was mixed with MeOH and different sample extracts were added. The diluted extract was mixed with the DPPH–methanol reagent and the absorbance was determined periodically for four hours at 517 nm.

#### 2.4.2. ORAC Assay

The ORAC value was defined according to Nurul et al. [30] with some amendments. The ORAC test measures the capacity of the active compounds to scavenge peroxyl radicals produced by spontaneous α,α′-Azodiisobutyramidine dihydrochloride (AAPH) radical decomposition [30]. A specific quantity of herbal extracts was mixed with a 13 mM phosphate buffer and 80 mM fluorescein and incubated for 20 minutes at 37 °C. After the initial fluorescence value was recorded, 60 mM DPPH radical was added. Fluorescence was monitored with a microplate reader for 150 minutes (Fluostar Omega, BMG Labtech, Ortenberg, Germany). The net area under the fluorescein decay curve (AUC) was determined and ORAC values were calculated by comparing the AUC to that of Trolox as a standard.

#### 2.4.3. TEAC Assay

The antioxidant capacities of these five herbal extracts were measured through a modified TEAC assay [30]. The TEAC assay was based on the reduction of the 2,2’-azinobis(3-ethylbenzothiazoline-6-sulfonic acid)•^−^ radicals cation (ABTS•^−^) by the antioxidants present in the samples. The principle of this method is the decolouration of the radical cation by the transfer of electrons that neutralize the free radicals, which causes a change colour in the solution [29]. Appropriate dilutions of extract samples were prepared. The ABTS•^−^ radical cation (7 mM) was dissolved and potassium sulphate (24.24 mM) was added and the mixture was allowed to remain in the dark for up to 16 hours. The phosphate-buffered saline (PBS), 10 mM was incubated for 30 min prior to use at room temperature. The assay was performed by mixing the sample with the solution containing ABTS•^−^ radical cation and the absorbance was measured at 734 nm using UV-visible spectrophotometer (Shimadzu, Japan). All the results were expressed as mg Trolox Equivalent (TE)/L sample.

### 2.5. Preparation of Active Packaging

Semi-refined carrageenan (SRC) powder (2% w/w) and distilled water were mixed and heated up to 60 °C with continuous stirring. Then, glycerol (0.9% v/v based on dry weight of SRC powder) was added as a plasticizer with continuous stirring at 70 °C. The film solution was heated up to 80 °C and the temperature was maintained with constant stirring for 10 minutes. After dissolution, the film solution was divided into six parts, control (SRC), SRC + G0.9% incorporating *Persicaria minor* (PM) extract at concentrations of 0.4, 1.0, 2.0% w/w and 0.4% w/w BHA. Antioxidants (PM extract and BHA) were added with magnetic stirring at 80 °C. Then, 100 mL of film-forming solution was cast on a casting plate (13 cm diameter) and it was dried for 1 day at the temperature 40 ± 2 °C. Finally, the film was peeled off from the casting plates [31].

### 2.6. Scanning Electron Microscopy (SEM)

The morphology of the surface and cross-section of films was observed using a scanning electron microscope (JEOL, JSM-6460LV, Tokyo, Japan). Samples for surface morphology were mounted on a bronze stub by double-sided tape, while for the cross-section, samples were cut into half using a razor blade and were made to stand on double-sided tape using carbon ink. Then, all of the samples were coated with gold. The images were captured with an accelerating voltage of 15 kV [32].

### 2.7. Preparation of Meat Patties

The minced meat (i.e., sirloin cuts) was purchased from a local market in Kuantan, Pahang. The meat was partitioned into five batches and mixed gently for 2 min with 1.5% of NaCl to obtain a good distribution of additives throughout the meat. Each of the samples was moulded into smaller parts (each sample approximately 20 g) and wrapped with film: (i) non-wrapped, (ii) control (SRC only), (iii) SRC + G (SRC with 0.9% (w/w) glycerol), (iv) SRC + G+ PM0.4% (SRC with 0.9% (w/w) glycerol and 0.4% (w/w) PM), (v) SRC + G + PM1.0% (SRC with 0.9% (w/w) glycerol and 1.0% (w/w) PM), (vi) SRC + G + PM2.0% (SRC with 0.9% (w/w) glycerol and 2.0% (w/w) PM) and (vii) SRC + G + BHA0.4% (SRC with 0.9% (w/w) glycerol and 0.4% (w/w) BHA) and placed in sterilized trays. All samples were wrapped in the developed films and stored under refrigeration (4 ± 2 °C) for 14 days and the changes in meat patties were analysed by the thiobarbituric acid reactive substance assay (TBARS) and metmyoglobin assays, and by measuring pH values at day 0, 3, 6, 9 and 14.

### 2.8. Thiobarbituric Acid Reactive Substance (TBARS) Assay 

The TBARS method was applied to determine the amount of lipid degradation throughout storage as reported previously [33]. One gram of each sample was weighed in a tube and mixed with 3 g/L aqueous Ethylenediaminetetraacetic acid (EDTA). The sample was then immediately dissolved with 5 mL of thiobarbituric acid reagent (TBARS) using an Ultra-Turrax (IKA, Germany) at 4000 rpm for 3 min. All procedures were performed in the dark and all samples were kept in ice. The mixture was incubated in hot water at 97 ± 1 °C for 10 min and shaken for 1 min during the process. The liquid sample was filtered and cooled for 10 minutes. The absorbance value for each sample was measured with a spectrophotometer at 532 nm. The value of TBARS was calculated using a malondialdehyde (MDA) standard curve prepared with 1,1,3,3-tetraethoxypropane and analysed by linear regression. All results were reported as mg malondialdehyde per kg sample (mg MDA/kg sample). All measurements were performed in triplicate.

### 2.9. Percentage of Metmyoglobin

The metmyoglobin assay performed was based on the study by Xu et al. [34]. Five grams of meat patties were homogenized using a homogenizer (Ultra-Turrax, IKA, Germany) with 25 mL ice-cold 0.04 M phosphate buffer (pH 6.8) for 15 s while the speed setting was set at 7 rpm. The homogenized mixture was allowed to stand at 4 °C for 1 h and centrifuged with a high-speed freezing centrifuge (GI-20 G, Anke, Shanghai, China) for 20 min at 4 °C. The absorbance of the filtered supernatant was read with a spectrometer (Fluostar Omega, BMG Labtech, Germany) at 572, 565, 545 and 525 nm. All measurements were performed in triplicate. The metmyoglobin percentage was calculated using the formula:

(1) $$MetMb\left(\%\right)=\left[-2.514\left(\frac{A572}{A525}\right)+0.777\left(\frac{A565}{A525}\right)+0.8\left(\frac{A545}{A525}\right)+1.098\right]\times 100$$

### 2.10. pH Measurement

The pH of the meat patties was measured directly on each sample using a GLP 21 pH meter (Mettler-Toledo, Columbus, OH, USA). All measurements were performed in triplicate.

### 2.11. Statistical Analysis

All experiments were conducted in triplicate, where the results were expressed as mean ± standard deviation (SD) or standard error (SE). The data were analysed using SPSS Statistical 17.0. Statistical analysis was performed using one-way analysis of variance (ANOVA) to measure the mean values between the groups. The multiple correction using Bonferroni’s test was used to determine significant differences at *p* < 0.05.

## 3. Results and Discussion

### 3.1. Total Phenolic Content and Antioxidant Activity of Different Edible Plants

Table 1 shows the total phenolic content of all plant extracts. *Persicaria minor* and *Cosmos caudatus* exhibited the highest polyphenol contents compared to CA, PS and SP, with an average 1.62 mg GAE/L sample, whereas CA showed the lowest value of polyphenol content with a 1.29 mg GAE/L sample (*p* < 0.05). The literature reports that PM contains flavonoids such as rutin, catechin, quercetin, isorhamnetin and kaempferol, whereas pentacyclic triterpenoids including asiaticoside, brahmoside, asiatic acid and brahmic acid are the polycyclic compounds in CA extracts [35]. According to Syed Ab Rahman et al. [24], phenolic acids and flavonoids including quercetin and naringin contribute to the total phenolic content detected by the Folin–Ciocalteu method.

Three types of antioxidant activity tests were carried out to assess the ability of plant extracts to scavenge different free radicals. The scavenging activity of the extracts is a way of determining plant antioxidant properties. Table 1 shows the results of the DPPH assays for five plant leaf extracts, with PM showing the highest value of 719.89 mg TE/L sample (*p* < 0.05). As reported in Table 1, PM was found to possess the highest antioxidant content, followed by CC, CA, PS and SP (*p* < 0.05). The phenolic compounds including flavonoids present in the PM and the CC plants contributed to the DPPH value. 

Furthermore, the antioxidant activity of natural leaf extracts was also determined using the ABTS method. The PM and CA extracts exhibited no significant difference in the TEAC values (*p* > 0.05), whereas SP showed the lowest TEAC value, 9.872 mg TE/ L (*p* < 0.05), as shown in Table 1. The radical cation ABTS•^+^ is formed by oxidation of ABTS by potassium persulfate. A stable non-coloured diamagnetic compound is formed by electron transfer from an antioxidant [30]. According to Reihani and Easa [36], the presence of phenolic compounds in CC plant extracts determines the TEAC value. 

The ability of plant extracts to scavenge peroxyl radicals was demonstrated by the ORAC test. The ORAC value was significantly increased in the order: SP < PS < CA < CC < PM (*p* < 0.05), as shown in Table 1. The order of activity was consistent with the DPPH and TEAC values. To the best of our knowledge, this is the first study that has reported the antioxidant activity of PM extracts by using the ORAC method. Ethanol has commonly been used for the extraction of polyphenol compounds from natural plants. According to Mello and Hubinger [37], the correlation between the content of flavonoids and polyphenols and the antioxidant activities did not display any significant differences of the plants extraction using ethanol. Many authors have reported that polyphenols have an influence on antioxidant activity. In *Portuguese propolis* extracts, there was no correlation between the content of phenols, flavones and flavonols and antioxidant activity [38], but a high positive correlation between polyphenol content of guava fruit juices and fluorescence recovery after photobleaching (FRAP) value was reported by Thaipong et al. [39]. A high correlation between the FRAP and DPPH values of nectarines, peaches and plums has also been reported by Gil et al. [40]. Phenolic compounds are known as hydrophilic antioxidants with correlations between their concentration and antioxidant activity [39]. Hence, various methods can be used to determine antioxidant activity and these data indicate activity comparable with that of previous reports.

### 3.2. Film Morphology

**Figure 1:**
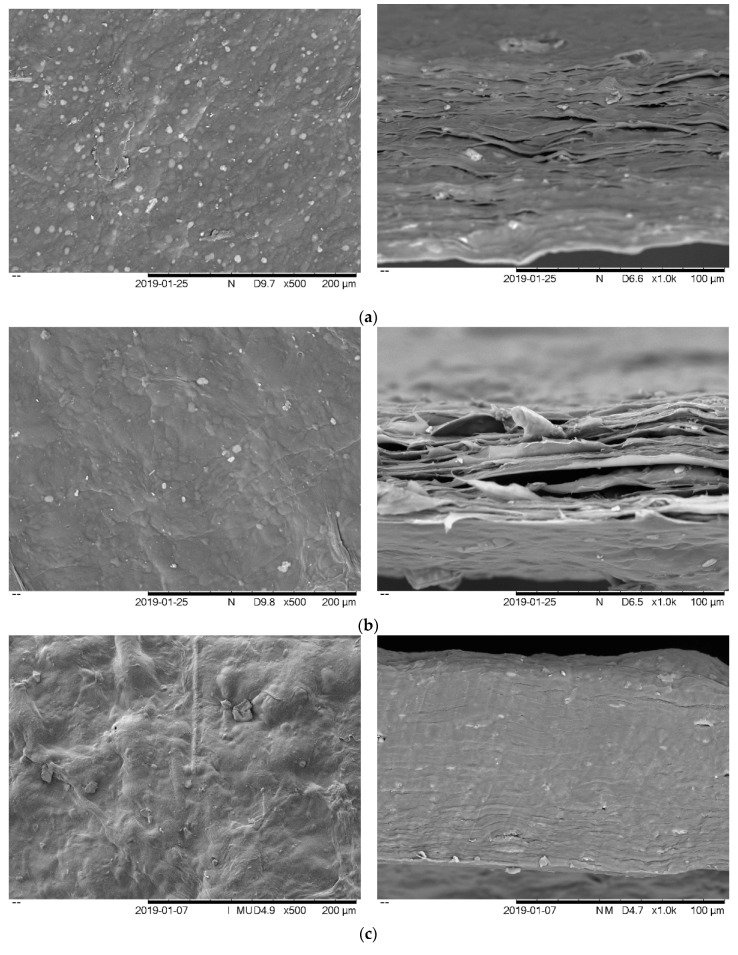

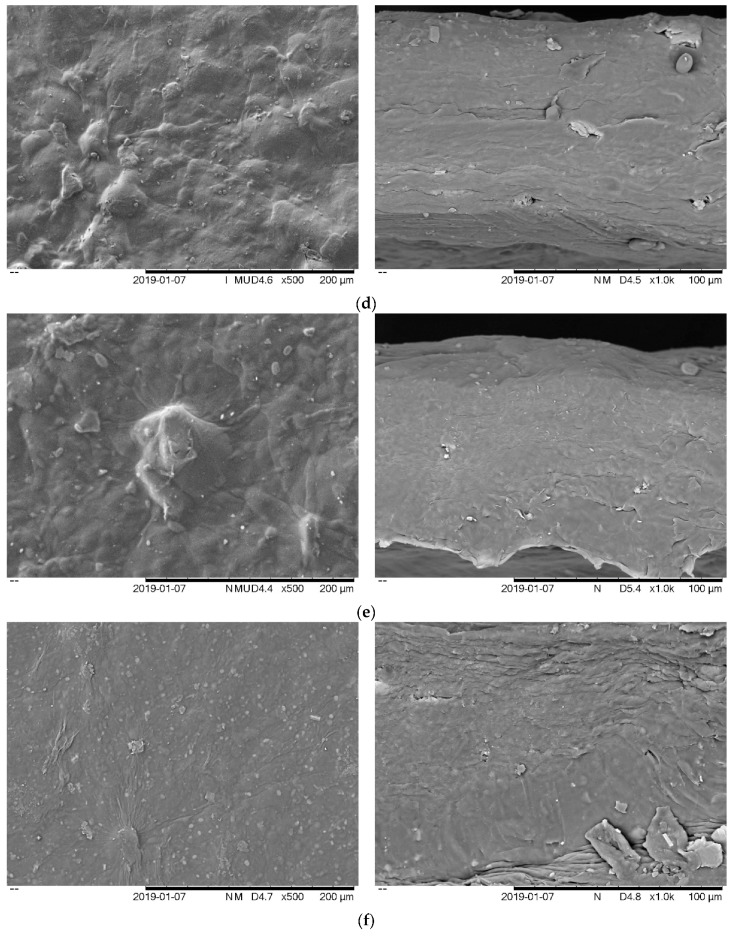
Surface and cross-section images of (**a**) SRC-control, (**b**) SRC + G, (**c**) SRC + G + PM0.4%, (**d**) SRC + G + PM1.0%, (**e**) SRC + G + PM2.0%, and (**f**) SRC + G + BHA films. SRC: Semi-refined carrageenan, G: Glycerol, PM: *Persicaria Minor,* BHA: Butylated hydroxyanisole.

Scanning electron microscopy (SEM) was used to determine the influence of film-forming agents (SRC, glycerol and *Persicaria minor*) and their distribution in the film matrix. The structural morphology of the surface and cross-sections of the SRC-control, SRC + G and SRC + G incorporated with different concentrations of PM (0.4%, 1.0%, 2.0% (w/w) and 0.4% w/w BHA) are shown in Figure 1. The neat carrageenan film (Figure 1a) showed a smooth and plane surface with no discontinuities or cleavage, whereas SRC + G (Figure 1b) had an inhomogeneous surface and bulges could be observed in a region of heterogeneity. This finding is similar to that reported in a previous study [41], in which chitosan film incorporating glycerol displayed heterogeneity within the film. These results are due to the hydrophilicity of the plasticizers where voids on the surface of the films could be formed due to the absorption of moisture. This layer showed that the film was not compact and was, thus, able to retain more water. Hence, this condition increased the moisture content, film solubility and flexibility of the films. Cross-sectional images revealed that un-plasticized films have homogeneous and compact microstructures, which are probably caused by strong cohesive forces that appear during the drying of the films. This finding is in agreement with the finding of a previous study [32], where the films prepared with neat carrageenan appeared to be smooth with no cleavage.

However, a considerable change in the carrageenan film microstructure was observed as the concentration of PM increased in the film. Certain regions of heterogeneity could be observed, which corresponded to the presence of entrapped extract droplets within the films. The consistency of the film topography was compromised and comparatively, non-uniform surface morphology was observed as the PM concentration increased. For SRC/PM 0.4% films (Figure 1c), small droplets were present embedded in the biopolymer matrix. As the PM concentration increased, the formation of larger droplets was observed, which may be attributed to the greater collision frequency within the droplets leading to the possibility of coalescence. This was possibly due to the addition of phenolic compounds which induce some discontinuities in the film matrix, due to the presence of hydrophilic compounds in the *Persicaria minor*.

### 3.3. TBARS Analysis of Stored Meat Patties

**Figure 2:**
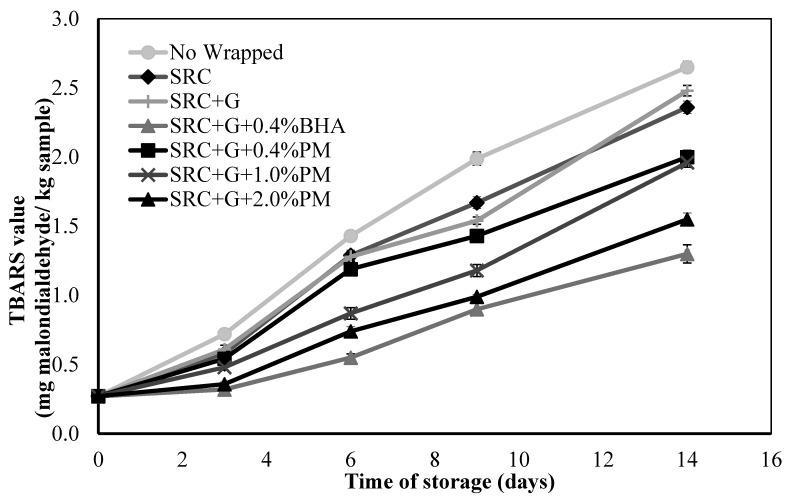
Changes in TBARS values (mg malondialdehyde/kg sample) in non-wrapped and control and samples containing 0.4, 1.0 and 2.0% (w/w) kesum leaf extract and 0.4% (w/w) BHA during 14 days of storage at 4 °C. Each sample was measured three times and the average standard deviation was less than 5% for each sample.

Figure 2 displays the lipid deterioration in different samples of meat patties during 2 weeks of storage. Thiobarbituric acid reactive substances (TBARS) were measured as an indicator of the secondary phase of lipid oxidation as mg malondialdehyde per kg of the sample. The deterioration of the secondary lipids would alter the flavour and contribute a rancid odour and unfavourable taste to the food [42]. The limit of the acceptable TBARS value for lipid oxidation was set at 1.0 mg malondialdehyde/kg sample [43]. The concentration of 0.4% BHA was added for comparison with the natural antioxidant as it represents a synthetic antioxidant that is widely used in industry. Figure 2 shows the TBARS values for the different concentrations of active film packaging of meat patties during 14 days of storage time. Overall, the highest concentration of PM and 0.4% BHA showed lower oxidation rates in meat patties compared to all other samples (*p* < 0.05). Lipid oxidation levels in meat patties increased during storage and the values followed the order: SRC + G + BHA0.4% < SRC + G + PM2.0% < SRC + G + PM1.0% < SRC + G + PM0.4% < SRC + G < Control < Non-wrapped (Figure 2). By comparison, non-wrapped meat patties showed the highest oxidation rate followed by the film without the addition of antioxidant compared to all samples with PM (*p* < 0.05). The samples of meat patties wrapped in different PM extract concentrations and wrapped with BHA film showed a significantly different TBARS value (*p* < 0.05) compared to the control. At day 9 of storage, samples packed with BHA and 2.0% PM extract exhibited the lowest TBARS value of below 1 mg malondialdehyde/kg sample.

A previous study showed that the total phenolic content of the PM extract was high with a value of 2800.6 ± 2.6 mg/100 g GAE, which is consistent with the high antioxidant activity shown by various assays [27]. The study also showed that flavonoids, phenolics and oxalic acids are present in PM. The antioxidant activity of a phenolic compound is related to the hydroxyl groups linked to the aromatic ring which are capable of donating hydrogen atoms with electrons and neutralizing free radicals. This mechanism blocks further degradation by active oxidation forming, for example, malondialdehyde (MDA), which can be measured using the TBARS method [44]. The most important intermediates in biological oxidation-reduction systems are free radicals. Biological oxidation occurs continuously, and its products are accumulated depending on age. Accumulated free radicals gradually cause cell and tissue injury and are closely related with the occurrence of age-related disorders. Cancer, inflammation, diabetes, cardiovascular diseases (CVD), neurological diseases, atherosclerosis, ageing and many other symptoms are linked to damage by free radicals. Antioxidants are substances capable of eliminating and scavenging excessive free radicals and repairing oxidative damage in biomolecules. They inhibit or delay the oxidation of biomolecules by preventing the initiation or propagation of oxidizing reactions in the chain [27]. The present study confirmed that the active packaging with the incorporation of PM extract demonstrated an antioxidant effect to inhibit lipid degradation in meat patties. 

The results are comparable to those found with pork burgers. In addition, avocado oil has been added directly to pork burgers and it showed a positive effect on burger preservation [45]. This study showed the protection of fat oxidation with good results. A previous study reported by Nurul et al. [30] confirmed that *Convolvulus arvensis* (CA) gelatin-based film displayed a powerful antioxidant effect to prevent lipid oxidation in muscle foods. The results show that CA extract can be used as a natural food antioxidant.

### 3.4. Percentage of Metmyoglobin

**Figure 3:**
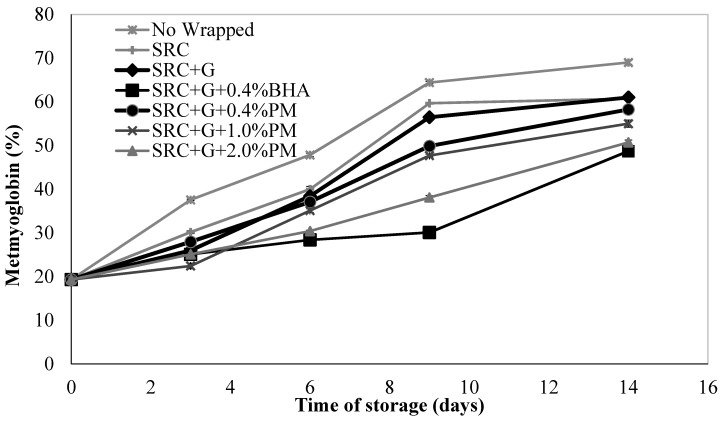
Changes in metmyoglobin percentage of non-wrapped, control and samples containing 0.4, 1.0 and 2.0% (w/w) of PM extract and 0.4% (w/w) BHA during 14 days storage at 4 ± 2 °C. Each sample was measured three times and the average standard deviation was less than 5% for each sample.

The colour of red meat is one of the important characteristics of its freshness and quality for consumers. The reduction of red colour by formation of metmyoglobin (brown colour) in meat is proportional to the oxidative degradation in meat throughout storage time. Figure 3 indicates the effect of the different concentrations of PM extract and BHA in packaging film on relative metmyoglobin percentage in meat patties. 

The formation of metmyoglobin was significantly increased in the following order: SRC + G + BHA0.4% < SRC + G + PM2.0% < SRC + G + PM1.0% < SRC + G + PM0.4% < SRC + G < Control (SRC) < Non-wrapped. Throughout the 14 days of storage, the % metmyoglobin increased with increased storage time, but the control showed the highest metmyoglobin concentration in comparison with all wrapped samples (*p* < 0.05). After 3 days storage, the film samples demonstrated no significant changes in colour (*p* > 0.05) but metmyoglobin formation increased rapidly from day 3 onwards (*p* < 0.05). Non-wrapped and control SRC showed greater metmyoglobin formation than other wrapped samples, with the concentration reaching more than 30% after 10 days storage, while the samples prepared with 2.0% (w/w) and 0.4% (w/w) BHA concentration displayed less brown colour development in the meat during 14 days storage (*p* < 0.05). A significant correlation between the metmyoglobin percentage and the redness was previously reported [46]. The colour decay and lipid oxidation depend on several factors, including storage time, type of packaging and test system. Lipid oxidation produces free radicals in meat which may initiate the reaction of oxidizing oxymyoglobin (red colour) to metmyoglobin (brown colour) resulting in meat discolouration during storage. Previous research has demonstrated a relationship between lipid oxidation and myoglobin oxidation or discolouration in meat products [34]. An adequate amount of antioxidant in the sample may delay metmyoglobin formation. The scavenging ability of antioxidant-treated samples can reduce metmyoglobin oxidation acting as scavengers of hydroxyl radicals produced by oxidation of oxymyoglobin [43]. There is no significant difference in the metmyoglobin concentration between samples prepared with 0.4% BHA and 2.0% PM extract and these samples demonstrated the lowest concentration of metmyoglobin compared to other samples (*p* < 0.05).

### 3.5. pH Measurement in Meat Patties

**Figure 4:**
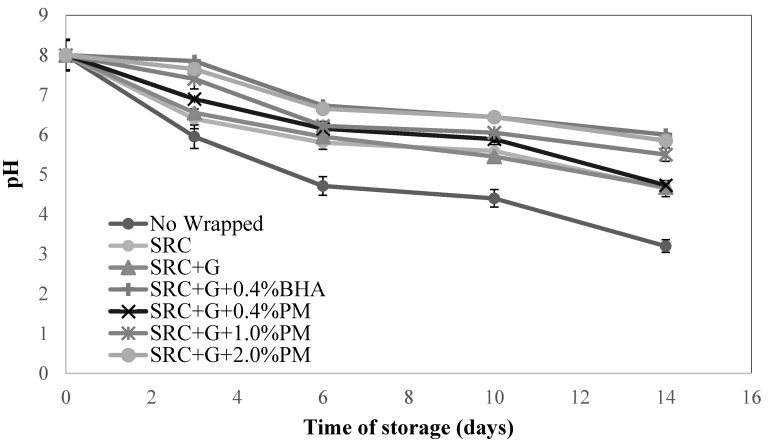
Change in pH of non-wrapped, control and samples containing 0.4, 1.0 and 2.0% (w/w) of PM extract and 0.4% (w/w) BHA during 14 days storage at 4 ± 2 °C. Each sample was measured in triplicate and the average standard deviation for each sample was less than 5%.

Figure 4 shows the changes in pH for SRC+G and samples with different concentrations of PM extract and BHA incorporated in the packaging. The pH value of the samples dropped over time with an inverse relationship to the % metmyoglobin and TBARS values. The pH of the meat samples decreased significantly in the order: SRC + G + BHA0.4% > SRC + G + PM2.0% > SRC + G + PM1.0% > SRC + G + PM0.4% > SRC + G > Control (SRC) > Non-wrapped. As indicated, the control showed the lowest pH compared to all samples containing antioxidant (*p* < 0.05), a fact that indicated the acidic condition in the meat. However, all samples with PM coating showed no significant differences in pH value throughout storage. From the result, it can be concluded that the addition of antioxidant improves pH values of meat samples. In the fat phase of the product, primary oxidation occurs quickly due to the formation of highly unstable hydroperoxides that easily break down. This process leads to the formation of acidic ketones, epoxides or organic acids and leads to pH changes [43,47]. Previously, active antioxidant packaging for meat products with oregano and rosemary extracts [48], barley husk extract [13] and citrus extract [49] has been prepared. The inclusion in the packaging material of natural extracts with antioxidant activity can protect food against lipid oxidation. To the best of our knowledge, this is the first study reporting the formulation of SRC-based films with glycerol as plasticizer and PM as natural antioxidant measured with meat as a food model.

## 4. Conclusions

This study demonstrated the total antioxidant activities and phenolic contents in five herbs found in Malaysia. As indicated by the results of the present study, Daun Kesum (*Persicaria minor*) displayed the highest antioxidant activity assessed by various antioxidant assays and in total phenolic content. A natural antioxidant from PM extract showed a positive effect on meat quality when incorporated in films with semi-refined carrageenan and glycerol as plasticizer. The SRC+G+PM2.0% showed the highest protection against lipid degradation in muscle foods and retained meat redness for longer during 14 days’ storage. Therefore, PM extract could be used as a source of natural antioxidants to be incorporated into SRC-based films to extend the food shelf life of the meat products. Further investigation is recommended to determine the effect on the sensorial properties of the meat patties stored wrapped in active packaging film.

## Figures and Tables

**Table 1:** Total phenolic content and antioxidant activity of different Malaysian herb extracts (Mean ± SE).

Assays	PM	CC	PS	CA	SP
TPC (mg GAE/L sample)	1.63 ± 0.02 ^a^	1.62 ± 0.02 ^a^	1.47 ± 0.01 ^b^	1.29 ± 0.01 ^c^	1.37 ± 0.02 ^d^
DPPH (mg TE/L sample)	719.89 ± 0.73 ^a^	680.27 ± 0.76 ^b^	576.24 ± 0.82 ^c^	600.09 ± 0.89 ^d^	478.34 ± 0.83 ^e^
ORAC (mg TE/L sample)	5.81 ± 0.05 ^a^	4.09 ± 0.03 ^b^	2.98 ± 0.12 ^c^	2.65 ± 0.08 ^d^	1.99 ± 0.05 ^e^
TEAC (mg TE/L sample)	27.16 ± 0.82 ^a^	11.90 ± 0.57 ^b^	16.75 ± 0.89 ^c^	27.59 ± 0.86 ^a^	9.87 ± 0.50 ^d^

PM: *Persicaria Minor*; CC: *Cosmos Caudatus*; PS: *Piper sarmentosum*; CA: *Centella asiatica*; SP: *Syzygium polyanthum.* Different letter (^a–e^) in the same row indicate significant difference (*p* < 0.05). Mean value *n* = 3 and the standard deviation for each assay was less than 5%. GAE: gallic acid equivalent; TE: Trolox equivalent.

## Abbreviations

SRC	Semi-refined carrageenan
DPPH	2,2-diphenyl-1-picrylhydrazyl
TEAC	Trolox equivalent antioxidant capacity
ORAC	oxygen radical absorbance capacity
PUFAs	polyunsaturated fatty acids
BHA	butylated hydroxyanisole
BHT	butylated hydroxytoluene
G	glycerol
CC	*Cosmos caudatus*
PS	*Piper sarmentosum*
PM	*Persicaria minor*
CA	*Centella asiatica*
SP	*Syzygium polyanthum*
TPC	total phenolic content
AUC	fluorescein decay curve
ABTS•^−^	2,2’-azinobis (3-ethylbenzothiazoline- 6-sulfonic acid)-radicals’ cation
TBARS	thiobarbituric reactive substance
EDTA	ethylenediaminetetraacetic acid
MDA	malondialdehyde
GAE	gallic acid equivalent
TE	Trolox equivalent
FRAP	fluorescence recovery after photobleaching
APPH	α,α′-Azodiisobutyramidine dihydrochloride
PBS	Phosphate-buffered saline
